# Morphology and magnetic properties of Fe_3_O_4_ nanodot arrays using template-assisted epitaxial growth

**DOI:** 10.1186/1556-276X-10-4

**Published:** 2015-01-06

**Authors:** Xiao-Fen Guan, Dan Chen, Zhi-Yong Quan, Feng-Xian Jiang, Chen-Hua Deng, Gillian Anne Gehring, Xiao-Hong Xu

**Affiliations:** Key Laboratory of Magnetic Molecules and Magnetic Information Materials of Ministry of Education and School of Chemistry and Materials Science, Shanxi Normal University, Linfen, 041004 China; Department of Physics and Astronomy, University of Sheffield, Hicks Building, Sheffield, S3 7RH UK

**Keywords:** Fe_3_O_4_, Nanodot arrays, PAA templates, Magnetic properties

## Abstract

Arrays of epitaxial Fe_3_O_4_ nanodots were prepared using laser molecular beam epitaxy (LMBE), with the aid of ultrathin porous anodized aluminum templates. An Fe_3_O_4_ film was also prepared using LMBE. Atomic force microscopy and scanning electron microscopy images showed that the Fe_3_O_4_ nanodots existed over large areas of well-ordered hexagonal arrays with dot diameters (*D*) of 40, 70, and 140 nm; height of approximately 20 nm; and inter-dot distances (*D*_int_) of 67, 110, and 160 nm. The calculated nanodot density was as high as 0.18 Tb in.^−2^ when *D* = 40 nm. X-ray diffraction patterns indicated that the as-grown Fe_3_O_4_ nanodots and the film had good textures of (004) orientation. Both the film and the nanodot arrays exhibited magnetic anisotropy; the anisotropy of the nanoarray weakened with decreasing dot size. The Verwey transition temperature of the film and nanodot arrays with *D* ≥ 70 nm was observed at around 120 K, similar to that of the Fe_3_O_4_ bulk; however, no clear transition was observed from the small nanodot array with *D* = 40 nm. Results showed that magnetic properties could be tailored through the morphology of nanodots. Therefore, Fe_3_O_4_ nanodot arrays may be applied in high-density magnetic storage and spintronic devices.

## Background

Fe_3_O_4_ has been extensively studied because of its high Curie temperature, and because it has been predicted to have a half metallicity and a full spin polarization [1, 2], these properties make Fe_3_O_4_ a promising material for applications in data storage and spintronic devices such as memories or magnetic sensors [3]. Epitaxially grown Fe_3_O_4_ nanofilms have been extensively investigated and have been reported to contain a type of natural growth defect, namely, antiphase boundaries (APBs) caused by strains on the film from mismatch between the substrate and the film [4, 5]. The presence of APBs leads to unusual magnetic properties of epitaxial Fe_3_O_4_ films, such as nonsaturation of magnetization in high magnetic fields [6]. Moreover, an epitaxial Fe_3_O_4_ nanodot array is a possible candidate to meet the requirements of ultrahigh-density storage and reduce the size of spintronic devices. In addition, the magnetic property dependence on nanodot size is worth investigating. However, to our knowledge, up to now, no studies on epitaxial Fe_3_O_4_ nanoarrays have been reported.

One popular top-down method, namely, the lithography technique, has been used to pattern films into nanoarrays [7, 8]. However, this top-down method has a complex procedure and is cost prohibitive for mass production. In addition, covalent oxides of Fe_3_O_4_ being fragile are easily damaged during etching. In another technique, Fe_3_O_4_ nanoparticles are synthesized first by chemical methods and then self-assemble on a substrate to form nanodot arrays [9, 10]. However, in this case, the particles have random orientations, easily aggregate, and have weak bonding force with the substrate, which make the properties quite different from those of epitaxial nanodot arrays.

Recently, porous anodized aluminum (PAA) templates have been used to fabricate large areas of metal and oxide nanodot arrays because of their low production cost, pore size controllability, and ease of fabrication, which is called the bottom-up method [11–15]. The PAA-based method is a direct approach for growing epitaxial nanodot arrays, in which the atoms passing through the pore directly arrive at the substrate. After removing the PAA template, an epitaxial growth of an ordered nanodot array is obtained.

In this paper, we used the bottom-up patterning technique, precisely controlled the fabrication parameters, and ultimately obtained large-area epitaxial Fe_3_O_4_ nanodot arrays on a single crystal substrate of SrTiO_3_. The magnetic property dependence on dot size and the morphology of nanoarrays were investigated and subsequently compared with their corresponding films. The density and size of the nanodots controlled by the PAA template could tailor magnetic properties including coercivity (*H*_c_), squareness, magnetic anisotropy, and Verwey transition temperature (*T*_v_).

## Methods

Ultrathin PAA templates with various pore diameters and inter-pore distances were prepared through a typical two-step anodization process [16, 17]. After the second anodization, the top surface of the PAA template was spin coated with a layer of polymethylmetacrylate (PMMA). The aluminum and thin nonporous barrier layer were then removed by immersing the template in an acid mixture of CuCl_2_ and HCl. A thin PMMA layer prevents the ultrathin ceramic membrane from undergoing mechanical deformations, such as folding, cracking, or ripping. After complete removal of aluminum, the template was transferred onto the SrTiO_3_ substrate and immersed in C_3_H_6_O at 60°C to dissolve the PMMA coating. Prior to the removal of PMMA, the templates were further thinned by immersing in H_3_PO_4_ for different durations to obtain the desired template thickness. In this work, three sizes of the PAA template with the same thickness of 200 nm were fabricated. The average pore diameter and inter-pore distance of the templates were (a) approximately 38 and approximately 67 nm, (b) approximately 67 and approximately 115 nm, and (c) approximately 137 and approximately 160 nm, respectively.

Fe_3_O_4_ was subsequently deposited on the PAA/SrTiO_3_ (001) substrate by laser molecular beam epitaxy (LMBE) using a KrF excimer laser (*λ* = 248 nm) with a repetition rate of 10 Hz and energy of 300 mJ on a ceramic target of Fe_3_O_4_. The PAA template was then removed using NaOH solution at 35°C, so that large-area Fe_3_O_4_ nanodot arrays were obtained. The substrate temperature was chosen between 400°C and 700°C, and the chamber oxygen pressure was 10^−4^ Pa. Some samples were annealed at 700°C *in situ* for 2 h before cooling to room temperature.

The crystal structure was characterized by X-ray diffraction (XRD) using Cu Kα radiation (*λ* = 0.15406 nm). The morphology of the PAA templates and nanodot arrays was investigated by scanning electron microscopy (SEM) and atomic force microscopy (AFM). Magnetization measurements were performed by a superconducting quantum interference device magnetometer (SQUID).

## Results and discussion

**Figure 1:**
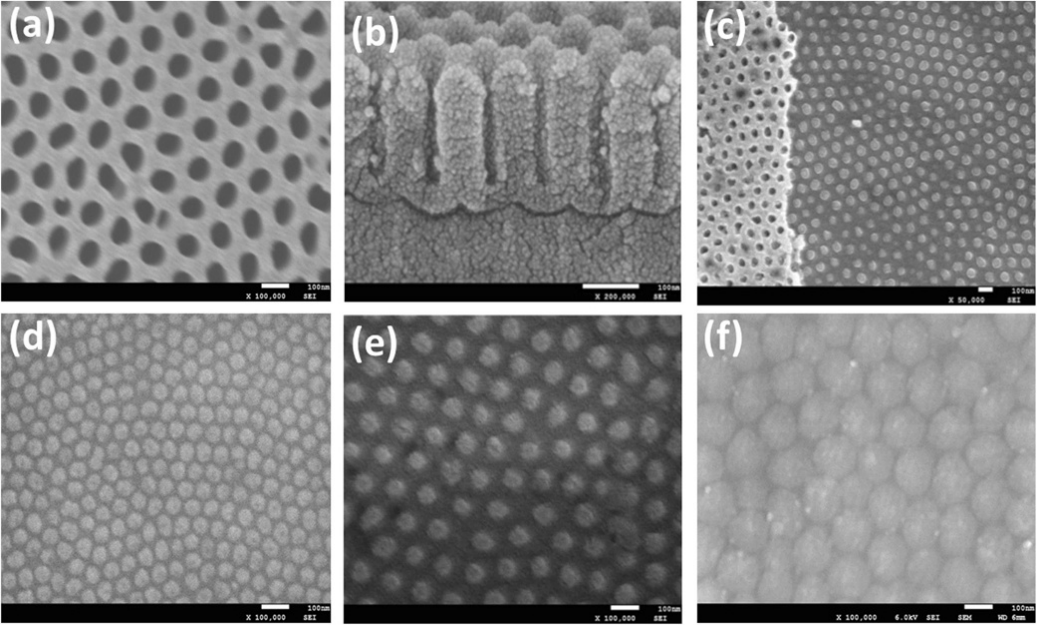
**SEM images for the PAA templates and Fe**_**3**_ **O**_**4**_ **dot arrays. (a)** Ultrathin PAA template, **(b)** cross section of the PAA, **(c)** Fe_3_O_4_ nanodot array together with a partially removed PAA template, and Fe_3_O_4_ nanodot arrays with various sizes - the average dot sizes and inter-dot periods are **(d)** approximately 40 and approximately 67 nm, **(e)** approximately 70 and approximately 115 nm, and **(f)** approximately 140 and approximately 160 nm, respectively.

Ultrathin PAA templates with pore diameters of 38, 67, and 137 nm were fabricated. Figure 1a shows a well-ordered array of circularly shaped holes with a pore diameter of 67 nm. Figure 1b is the cross section of this PAA template, with a thickness of approximately 200 nm. The high-quality ultrathin PAA template is the key in preparing epitaxial Fe_3_O_4_ nanodot arrays. When the PAA template was partially removed, the Fe_3_O_4_ nanodot arrays and the cover of the PAA template were clearly observed, as shown in Figure 1c. The resulting nanodot arrays have respective dot diameters (*D*) and inter-dot distances (*D*_int_) of approximately 40 and approximately 67 nm (Figure 1d), approximately 70 and approximately 115 nm (Figure 1e), and approximately 140 and approximately 160 nm (Figure 1f). These *D* were a slightly larger than the pore diameter of PAA, which may be due to the diffusion of atoms during deposition and annealing. The nanodot density was estimated using the following equation [15]: . As shown in Figure 1d, the dot density can be as high as 0.18 Tb in.^−2^, using Fe_3_O_4_ nanodot arrays with *D* and *D*_int_ down to 40 and 67 nm.

**Figure 2:**
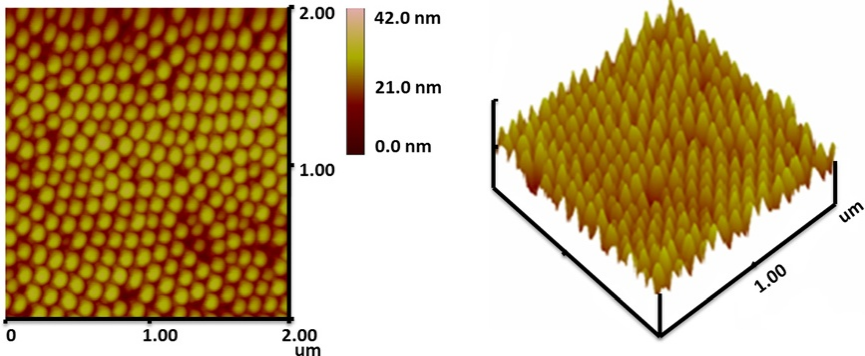
**AFM images of Fe**_**3**_ **O**_**4**_ **dot array with** ***D*** **= 70 nm.**

AFM images of the Fe_3_O_4_ nanodot array with *D* of approximately 70 nm and *D*_int_ of approximately 115 nm are shown in Figure 2. These images confirm that nanodots have a well-ordered hexagonal arrangement in agreement with the SEM results. Although the nanodots and the film were prepared using the same parameters, the average dot height of Fe_3_O_4_ is around 20 nm, which is slightly lower than the thickness (24 nm) of the film. Possibly, some of the atoms cannot arrive at the bottom of the pore, but rather are deposited on the surface of the pore wall and disappeared during the removal of the PAA templates.

**Figure 3:**
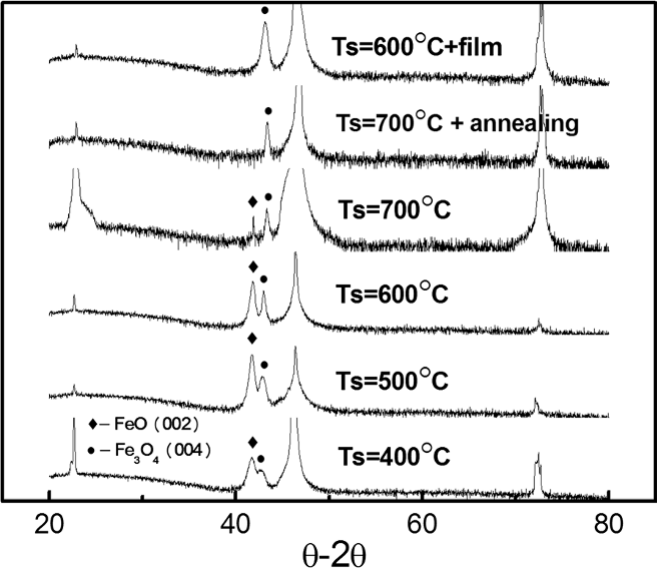
**XRD patterns for the film and dot arrays.** The sample of *D* = 70 nm and film deposited at various substrate temperatures and after annealing. Except for the peaks of FeO (002) and Fe_3_O_4_ (004), the rest are peaks of the substrate.

The *θ*-2*θ* scans of the series of film and nanodot arrays deposited at different substrate temperatures (*T*_s_), as well as the scans after annealing, are shown in Figure 3. The XRD patterns of the nanodot arrays without *in situ* annealing showed peaks that correspond to FeO (002), as well as the Fe_3_O_4_ (004) reflections. The intensity of the Fe_3_O_4_ (004) peaks increased, whereas the FeO (002) peaks decreased with an increase of *T*_s_. These trends indicate that high substrate temperature favors the formation of the Fe_3_O_4_ (004) phase. The peak of FeO (002) disappeared after 2 h of *in situ* annealing at 700°C. In this condition, epitaxial growth of Fe_3_O_4_ nanodots with (004) orientation was obtained. The Fe_3_O_4_ (004) film could have epitaxial growth at a *T*_s_ of 600°C. Clearly, epitaxial growth of Fe_3_O_4_ nanodots is more difficult to achieve compared with that of the corresponding film. This difficulty may be attributed to the following reasons: (1) The edge of the nanosized pore may hinder deposition resulting in some of the atoms losing part of their energy so that atom hopping time and length are reduced, which is unfavorable for epitaxial growth. (2) The atoms enter the nanosized pores rather than on the flat substrates. Part of the atoms neighboring the wall of the pore obviously cannot move to the normal direction of the wall. This limitation also influences epitaxial growth. (3) The atoms in the pores may be separated by PAA from the activated oxygen atoms. Meanwhile, the energy for FeO formation was lower than that of Fe_3_O_4_ [18], so part of the Fe_3_O_4_ may have been reduced. From the above analysis, we can conclude that with the aid of high *T*_s_ and *in situ* annealing, the deposited atoms which gained more thermal dynamic energy finally resulted in the good quality of Fe_3_O_4_ nanodot arrays. Following the optimal parameters of a 70-nm nanodot array, with *D* = 40, 140-nm-sized arrays all grow at *T*_s_ = 700°C and 2 h of *in situ* annealing at 700°C.

**Figure 4:**
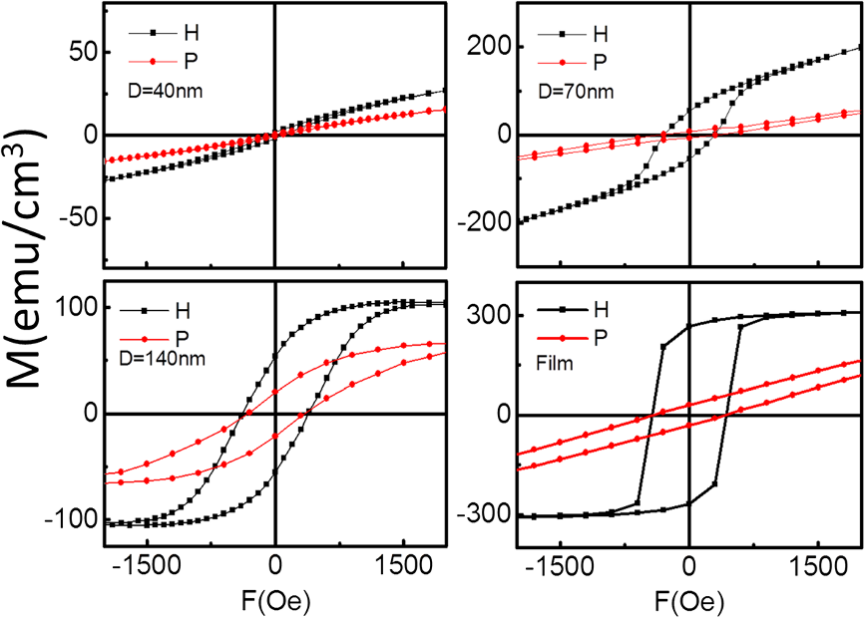
**Hysteresis loops of Fe**_**3**_ **O**_**4**_ **film and dot arrays with** ***D*** **= 40, 70, and 140 nm.** The H and P means the hysteresis loops of the samples measured at in-plane and out-of-plane to the film directions.

**Figure 5:**
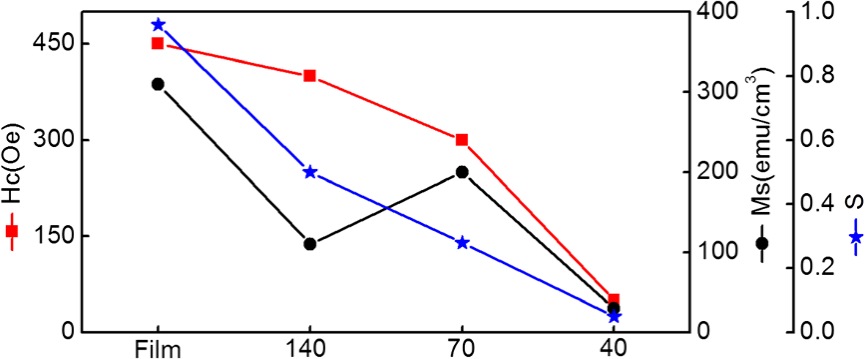
**Variations in** ***H***_**c**_ **,** ***M***_**s**_ **, and** ***S*** **as functions of Fe**_**3**_ **O**_**4**_ **film and different nanodot diameters.**

In-plane (H) and out-of-plane (P) room-temperature hysteresis loops of the Fe_3_O_4_ nanodot arrays and the film acquired are shown in Figure 4. The *H*_c_, the squareness factor (*S*), and the saturation magnetization (*M*_s_) of nanodot arrays and the film are shown in Figure 5. These show that the magnetic in-plane easy-axis anisotropy was observed, and the anisotropy is reduced with the dot size. Possibly, the magnetostrictive anisotropy was greatly reduced by the fast relaxation strain from the dot edges, whereas the shape anisotropy and crystalline anisotropy were released by forming noncontinuous dots [19]. As a result, the smallest nanodots exhibited a weak magnetic anisotropy.

The *M*_s_ of the film (310 emu/cm^3^) is smaller than that of the bulk (480 emu/cm^3^), which was attributed to the presence of APBs. The lattice constant (0.8398 nm) of the Fe_3_O_4_ film grown on SrTiO_3_ (0.3905 nm) is more than twice that of the substrate, indicating that the film undergoes a compressive strain. This strain induces some defects such as dislocations or APBs during film growth. Presences of APBs are the main reason for the lower saturation magnetic value compared with the bulk Fe_3_O_4_ [20]. Moreover, the *M*_s_ of the nanodot arrays is smaller than that of the film. This characteristic could be explained by the accumulation of spin canting around the nanoparticle surface and the exchange coupling between the nanodots that reduce magnetic moment [21].

From Figure 5, the in-plane hysteresis loop of the film displayed an almost rectangular shape with *S* = 0.96 and a large *H*_c_ of 450 Oe, which is better than that reported for Fe_3_O_4_ films with comparable thickness [6]. The sample with *D* = 140 nm had a large *H*_c_ of 400 Oe and was easily saturated. The magnetization reversal may be dominated by the domain wall movement, similar to the film. However, the *S* of this sample is 0.50, which was half that of the film. The reduced *S* may be due to magnetization relaxation from dot edges. The sample with *D* = 70 nm exhibited smaller *S* = 0.28, large *H*_c_ of 300 Oe, and unsaturation at an external field of 2,000 Oe. At *S* = 0.05, *H*_c_ dropped to 50 Oe and unsaturation of the sample with *D* = 40 nm exhibited more or less soft magnetic properties similar to those of the bulk. The sample with *D* = 40 nm may reverse their magnetization by coherent rotation because of a single domain size of 38 nm for Fe_3_O_4_ [22]. On the other hand, the sample with *D* = 70 nm with its relatively high *M*_s_ and *H*_c_ maybe in a transition state so that reversal is partly due to wall movement, impeded by defects, and rotation, which should be further investigated in the future.

**Figure 6:**
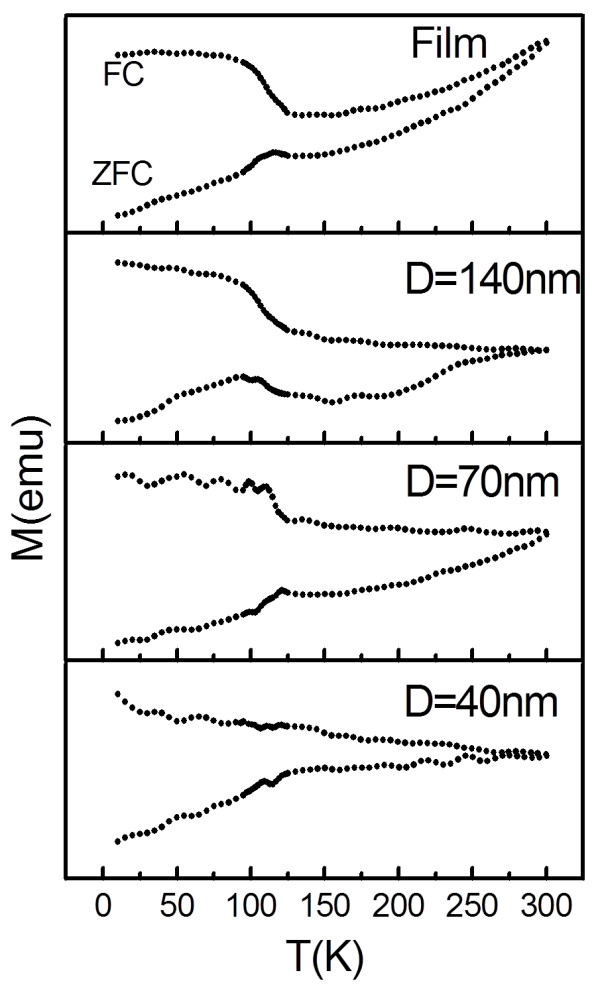
**FC-ZFC curves of Fe**_**3**_ **O**_**4**_ **film and dot arrays with** ***D*** **= 40, 70, and 140 nm.**

Plots of field cooling-zero field cooling (FC-ZFC) magnetization versus temperature between 10 and 300 K are shown in Figure 6. A magnetic field of 50 Oe had been applied on the plane during measurement. The *T*_v_ obtained was around 120 K for the Fe_3_O_4_ film and arrays with *D* = 140 and 70 nm, close to that of the bulk Fe_3_O_4_ [23]. However, *T*_v_ was undetectable at *D* = 40 nm. Normally, the presence of *T*_v_ is used as an evidence that the sample has a perfect stoichiometry of Fe:O = 3:4. The identity of the sample with *D* = 40 nm had been confirmed to be Fe_3_O_4_ based on the XRD pattern. The undetected *T*_v_ may be attributed to its small particle size and relatively large inter-particle spacing. The hopping energy barrier between different Fe sites within the nanodot was relatively small compared with the inter-nanodot tunneling barrier, so no *T*_v_ was observed in the sample [24].

## Conclusions

In summary, well-ordered large-area arrays of epitaxial Fe_3_O_4_ nanodots were obtained on the SrTiO_3_ (100) substrate by LMBE at an elevated growth temperature (700°C) using ultrathin PAA templates as shadow templates. An Fe_3_O_4_ film was also prepared using LMBE. The AFM and SEM images showed that Fe_3_O_4_ nanodot arrays had large areas with well-ordered hexagonal arrays with *D* of 40, 70, and 140 nm; height of around 20 nm; and *D*_int_ of 67, 115, and 160 nm. The nanodot density can be calculated to be as high as 0.18 Tb in.^−2^ when *D* = 40 nm. The film or nanodot array exhibited magnetic anisotropy. The anisotropy of the arrays and the *S* weakened with decreasing dot size. Magnetization reversal may be dominated by the domain wall movement of the film and of the sample with *D* = 140 nm, while the sample with *D* = 40 nm may reverse their magnetization by coherent rotation because of its single domain size of 38 nm. On the other hand, the sample with *D* = 70 nm exhibited smaller *S*, large *H*_c_, and unsaturation at the external field of 2,000 Oe. It demonstrated that the sample with *D* = 70 nm may undergo a magnetization reversal transition, which should be further investigated. The *T*_v_ values of the film and the samples with large dots (*D* = 70 and 140 nm) were close to those of the bulk, whereas the *T*_v_ of the small dot sample with *D* = 40 nm was undetected. Based on the above analysis, magnetic anisotropy, *S*, and *T*_v_ of Fe_3_O_4_ could be tailored by the morphology of nanodots. To the best of our knowledge, no study has shown epitaxial Fe_3_O_4_ nanodots to date. These results facilitate a deeper understanding of the micromagnetization inside nanostructures of Fe_3_O_4_, and the output could be a candidate for ultrahigh-density data storage and spintronic devices.
